# Supplementary material for: Effects of relational and instrumental messaging on human perception of rattlesnakes
Source: PLoS One. 2024 Apr 17;19(4):e0298737. doi: 10.1371/journal.pone.0298737 (PMC11023442; doi:10.1371/journal.pone.0298737)
Supplement: S1 File — (PDF) [file pone.0298737.s001.pdf]

Perception of Rattlesnakes

This survey is expected to take no more than 15 minutes. You will be prompted to enter for a chance to win an iPad once completing the survey. Participating in the giveaway is optional. You may only complete the survey once.

* Indicates required question

Informed Consent

Evaluating Public Perception of Rattlesnakes

Principal Investigator: Erin Allison, Graduate Assistant, West Liberty University
Research Advisor: Zachary Loughman, Associate Professor of Biology, West Liberty University

Important Information about the Research Study: You are invited to participate in a research study. In order to participate, you must be an adult 18 years or older. Taking part in this research project is voluntary. The purpose of this study is to evaluate the public's perception of rattlesnakes. If you agree to take part in this study, you will be asked to provide demographic information, past experience with rattlesnakes, exposed to an educational rattlesnake video, and asked about your perception of rattlesnakes. We expect this to take about 15 to 20 minutes.

How could you benefit from this study? You might benefit from being in this study because you will be presented with the opportunity to be entered for a chance to win an iPad. We plan to publish the results of this study. To protect your privacy, we will not include any information that could directly identify you. We will protect the confidentiality of your research records by taking additional measures to password protect the data. Your email address and any other information that can directly identify you will be stored separately from the data collected as part of the project. There is minimal risk associated with participating in this study.

Information Collected from Study: It is possible that other people may need to see the information we collect about you. These people work for West Liberty University, and government offices that are responsible for making sure the research is done safely and properly. We will not keep your research data to use for future research or other purposes. Your email address and other information that can directly identify you will be deleted from the research data collected as part of the project. We may share your research data with other investigators without asking for your consent again, but it will not contain information that could directly identify you.

Incentive for Participation: You will receive the opportunity to enter for a chance to win an iPad as an incentive for participating in this study. At the end of the survey, you will be asked for your email address which will be used to contact the randomly selected winner. You will not receive any additional emails pertaining to this study. You will only be contacted if randomly selected to win an iPad. Your email will not be shared with anyone. Participating in the incentive giveaway is optional. You are not required to share your email address. If the randomly selected winner does not respond in 14 business days, a new winner will be chosen. The iPad will be mailed to winner's mailing address at no cost to them.

Your Participation is Voluntary: There are no costs to you to participate in this study. All study related materials will be provided to you. It is totally up to you to decide to be in this research study. Participating in this study is voluntary. Even if you decide to be part of the study now, you may change your mind and stop at any time. You do not have to answer any questions you do not want to answer. If you decide to withdraw before this study is completed, your data may not be used in this study.

Contact Information for Study Team: If you have questions about this research, you may contact Erin Allison; eballison@westliberty.edu or Zac Loughman; zloughman@westliberty.edu

Contact Information for your Right as a Research Participant: If you have questions about your rights as a research participant, or wish to obtain information, ask questions, or discuss any concerns about this study with someone other than the researcher(s), please contact the WLU IRB or the WLU Research Integrity Officer
irb@westliberty.edu
Research Integrity Officer 304-336-8004 (Office of the Provost WLU)
West Liberty University
[illegible]

208 University Drive
West Liberty, WV 26074

1. Your Consent: By checking this box, you are agreeing to be in this study. Make sure you understand what the study is about before you agree. We will give you a copy of this document for your records. We will keep a copy with the study records. If you have any questions about the study after you sign this document, you can contact the study team using the information provided above. *

   Check all that apply.

   - [ ] I understand what the study is about and I agree to take part in this study.

Pre-Survey

Please answer the following questions regarding your perception of rattlesnakes.

2. Rattlesnakes are fascinating animals.

Strongly agree=1 Agree=2 Neutral=3 Disagree=4 Strongly disagree=5

Mark only one oval.

Strongly Agree

1 ⬭

2 ⬭

3 ⬭

4 ⬭

5 ⬭

Strongly Disagree

3. Rattlesnakes are not important to the ecosystem.

Strongly agree=1 Agree=2 Neutral=3 Disagree=4 Strongly disagree=5

Mark only one oval.

	Strongly Agree
1	◯
2	◯
3	◯
4	◯
5	◯
	Strongly Disagree

4. I would like to encounter a rattlesnake in the wild.

Strongly agree=1 Agree=2 Neutral=3 Disagree=4 Strongly disagree=5

Mark only one oval.

	Strongly Agree
1	◯
2	◯
3	◯
4	◯
5	◯
	Strongly Disagree

5. Rattlesnakes are generally misunderstood by humans.

Strongly agree=1 Agree=2 Neutral=3 Disagree=4 Strongly disagree=5

Mark only one oval.

Strongly Agree

1 ⬭

2 ⬭

3 ⬭

4 ⬭

5 ⬭

Strongly Disagree

6. If I came across a rattlesnake, I feel like it would harm me.

Strongly agree=1 Agree=2 Neutral=3 Disagree=4 Strongly disagree=5

Mark only one oval.

Strongly Agree

1	⬭
2	⬭
3	⬭
4	⬭
5	⬭

Strongly Disagree

7. It is acceptable to injure a rattlesnake because they could injure people.

Strongly agree=1 Agree=2 Neutral=3 Disagree=4 Strongly disagree=5

Mark only one oval.

Strongly Agree

1 ⬭

2 ⬭

3 ⬭

4 ⬭

5 ⬭

Strongly Disagree

8. What is your overall perception of rattlesnakes?

Mark only one oval.

- Positive
- Somewhat positive
- Neutral
- Somewhat negative
- Negative

9. Which statement best fits your current perception of rattlesnakes?

Mark only one oval.

- I have an affinity for rattlesnakes.
- I find rattlesnakes interesting.
- I am indifferent towards rattlesnakes.
- I do not find rattlesnakes interesting.
- I have a phobia of rattlesnakes.

Demographic Survey

Please fill out the following demographic questions. Answers will remain confidential.

10. Which continent do you live in? *

Mark only one oval.

- ⬭ North America
- ⬭ South America
- ⬭ Europe
- ⬭ Australia
- ⬭ Oceania
- ⬭ Asia
- ⬭ Africa

11. If you live in the United States, what is your State or Territory?

If not, leave blank

Mark only one oval.

- ALABAMA
- ALASKA
- AMERICAN SAMOA
- ARIZONA
- ARKANSAS
- CALIFORNIA
- COLORADO
- CONNECTICUT
- DELAWARE
- DISTRICT OF COLUMBIA
- FLORIDA
- GEORGIA
- GUAM
- HAWAII
- IDAHO
- ILLINOIS
- INDIANA
- IOWA
- KANSAS
- KENTUCKY
- LOUISIANA

- [ ] MAINE
- [ ] MARYLAND
- [ ] MASSACHUSETTS
- [ ] MICHIGAN
- [ ] MINNESOTA
- [ ] MISSISSIPPI
- [ ] MISSOURI
- [ ] MONTANA
- [ ] NEBRASKA
- [ ] NEVADA
- [ ] NEW HAMPSHIRE
- [ ] NEW JERSEY
- [ ] NEW MEXICO
- [ ] NEW YORK
- [ ] NORTH CAROLINA
- [ ] NORTH DAKOTA
- [ ] OHIO
- [ ] OKLAHOMA
- [ ] OREGON
- [ ] PENNSYLVANIA
- [ ] PUERTO RICO
- [ ] RHODE ISLAND
- [ ] SOUTH CAROLINA
- [ ] SOUTH DAKOTA

- TENNESSEE
- TEXAS
- UTAH
- VERMONT
- VIRGINIA
- VIRGIN ISLANDS
- WASHINGTON
- WEST VIRGINIA
- WISCONSIN
- WYOMING

12. What is your age (integer only)? *

____

13. Which gender do you identify with? *

Mark only one oval.

- Male
- Female
- Non-binary
- None of the Above
- Prefer not to say

14. Which of the following best describes where you live?

Mark only one oval.

- Urban
- Suburban
- Rural or Remote
- Not sure

15. What is the highest level of education you have completed? *

Mark only one oval.

- Some high school
- High school Diploma/GED
- Associates Degree
- Trade School
- Bachelor's Degree
- Master's Degree
- Ph.D or higher
- Prefer not to say

16. Please specify your religion.

Mark only one oval.

- Agnosticism
- Atheism
- Buddism
- Christianity
- Hinduism
- Islam
- Judaism
- Spiritual
- Prefer not to say

Experience with Rattlesnakes

Please answer the following questions about your experiences with rattlesnakes.

17. Do rattlesnakes occur in your local area? *

Mark only one oval.

- Yes
- No
- Not sure

18. Have you ever encountered a rattlesnake in nature? *

Mark only one oval.

- Yes
- No
- Not sure

19. Have you ever encountered a captive rattlesnake at a zoo or nature center? *

Mark only one oval.

- Yes
- No
- Not sure

20. Have you, a friend, or your pet ever been bitten by a venomous snake? *

Mark only one oval.

- Yes
- No
- Not sure

21. If you answered yes, check all that apply:

If you answered no, leave blank

Check all that apply.

- [ ] Yourself
- [ ] Friend
- [ ] Pet

Participant Choice

This section is used to ensure proper participant sampling.

22. Please select the first image displayed below. *

Mark only one oval.

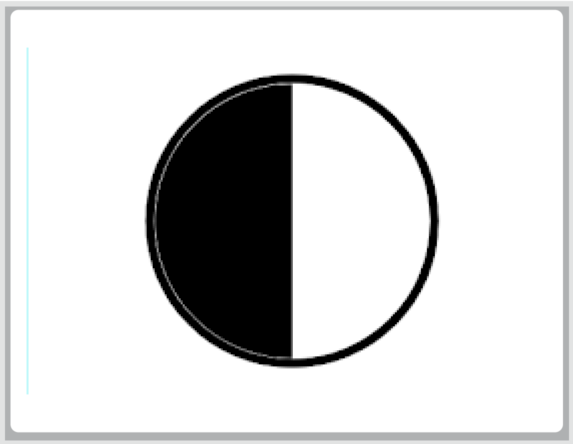

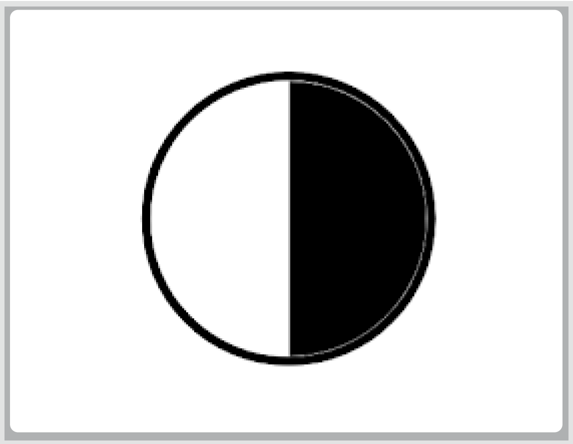

( ) . *Skip to question 23*

( ) , *Skip to question 24*

Skip to question 22

A Short Video

Please watch this short video about rattlesnakes before moving forward.

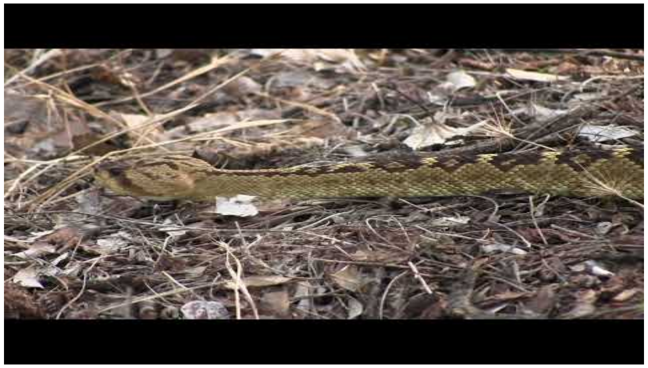 http://youtube.com/watch?v=5fZnHWeRifg

23. Please confirm that you watched a video about rattlesnakes. *

Check all that apply.

☐ Yes, I watched a video.

Skip to question 25

A Short Video

Please watch this short video about rattlesnakes before moving forward.

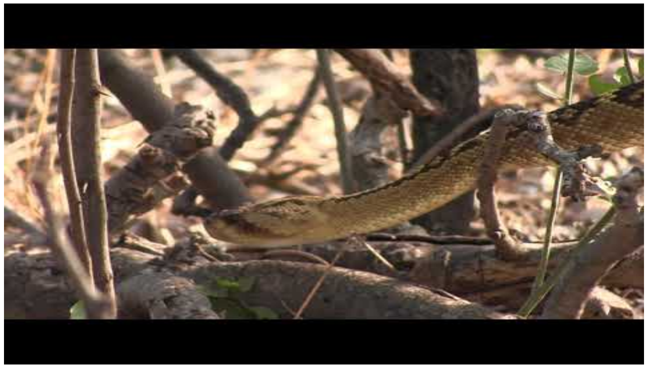 http://youtube.com/watch?v=FUxg4cY-c4I

24. Please confirm that you watched a video about rattlesnakes. *

Check all that apply.

☐ Yes, I watched a video.

Skip to question 25

Post-Survey

Please answer the following questions regarding your perception and knowledge of rattlesnakes.

25. Rattlesnakes are fascinating animals.

Strongly agree=1 Agree=2 Neutral=3 Disagree=4 Strongly disagree=5

Mark only one oval.

	Strongly Agree
1	◯
2	◯
3	◯
4	◯
5	◯
	Strongly Disagree

26. Rattlesnakes are not important to the ecosystem.

Strongly agree=1 Agree=2 Neutral=3 Disagree=4 Strongly disagree=5

Mark only one oval.

Strongly Agree

1 ⬭

2 ⬭

3 ⬭

4 ⬭

5 ⬭

Strongly Disagree

27. I would like to encounter a rattlesnake in the wild.

Strongly agree=1 Agree=2 Neutral=3 Disagree=4 Strongly disagree=5

Mark only one oval.

Strongly Agree

1 ⬭

2 ⬭

3 ⬭

4 ⬭

5 ⬭

Strongly Disagree

28. Rattlesnakes are generally misunderstood by humans.

Strongly agree=1 Agree=2 Neutral=3 Disagree=4 Strongly disagree=5

Mark only one oval.

Strongly Agree

1

2

3

4

5

Strongly Disagree

29. If I came across a rattlesnake, I feel like it would harm me.

Strongly agree=1   Agree=2   Neutral=3   Disagree=4   Strongly disagree=5

Mark only one oval.

	Strongly Agree
1	◯
2	◯
3	◯
4	◯
5	◯
	Strongly Disagree

30. It is acceptable to injure a rattlesnake because they could injure me.

Strongly agree=1 Agree=2 Neutral=3 Disagree=4 Strongly disagree=5

Mark only one oval.

	Strongly Agree
1	⬭
2	⬭
3	⬭
4	⬭
5	⬭
	Strongly Disagree

31. What is your overall perception of rattlesnakes?

Mark only one oval.

- Positive
- Somewhat positive
- Neutral
- Somewhat negative
- Negative

32. Which statement best fits your current perception of rattlesnakes?

Mark only one oval.

- I have an affinity for rattlesnakes.
- I find rattlesnakes interesting.
- I am indifferent towards rattlesnakes.
- I do not find rattlesnakes interesting.
- I have a phobia of rattlesnakes.

33. Additional comments regarding your perception of rattlesnakes. (optional)

This content is neither created nor endorsed by Google.

Google Forms
