# Supplementary material for: Effects of relational and instrumental messaging on human perception of rattlesnakes
Source: PLoS One. 2024 Apr 17;19(4):e0298737. doi: 10.1371/journal.pone.0298737 (PMC11023442; doi:10.1371/journal.pone.0298737)
Supplement: S1 Text — (DOCX) [file pone.0298737.s002.docx]

**S1 Text. The demographic questionnaire with items regarding individuals socio-demographic information and prior experience with rattlesnakes.** The demographic questionnaire was embedded after the pre-Rattlesnake Perception Test and before viewing a randomly selected video.

| Socio-demographic Question | Answer Choices |
| --- | --- |
| Which continent do you live in? | North America, South America, Europe, Australia, Oceania, Asia, Africa |
| If you live in the United States, what is your State or Territory? | All 50 U.S. states, District of Columbia, Guam, Puerto Rico, American Samoa, Virgin Islands |
| What is your age (integer only)? | Self-reported fill-in-the-blank |
| Which gender do you identify with? | Male, Female, Non-binary, None of the above, Prefer not to say |
| Which of the following best describes where you live? | Urban, Suburban, Rural or remote, Not sure |
| What is the highest level of education you have completed? | Some high school, High school diploma/GED, Associates degree, Trade school, Bachelor’s degree, Master’s degree, Ph.D. or higher, Prefer not to say |
| Please specify your religion | Agnosticism, Atheism, Buddhism, Christianity, Hinduism, Islam, Judaism, Spiritual, Prefer not to say |
| Experience with Rattlesnakes Questions | Answer Choices |
| Do rattlesnakes occur in your local area? | Yes, No, Not sure |
| Have you ever encountered a rattlesnake in nature? | Yes, No, Not sure |
| Have you ever encountered a captive rattlesnake at a zoo or nature center? | Yes, No, Not sure |
| Have you, a friend, or your pet ever been bitten by a venomous snake? | Yes, No, Not sure |
| If you answered yes, check all that apply: | Yourself, Friend, Pet |
