# Supplementary material for: Effects of relational and instrumental messaging on human perception of rattlesnakes
Source: PLoS One. 2024 Apr 17;19(4):e0298737. doi: 10.1371/journal.pone.0298737 (PMC11023442; doi:10.1371/journal.pone.0298737)
Supplement: S2 Text — (DOCX) [file pone.0298737.s003.docx]

**S2 Text.** **Compiled list of information on social media sites used to disseminate the Rattlesnake Survey.**

I. The survey itself resided on its on Facebook page witch since has been removed. The title of the page was “Rattlesnake Survey”

---------------------------------------------------------------------------------------------------------------------

II. Facebook pages/Sites where the Rattlensnake Survey was shared:

Gardening in Ohio

[(2) Gardening in Ohio | Facebook](https://www.facebook.com/groups/132988540048075)

Gardening Tips for Beginners

[(2) Gardening Tips for Beginners | Facebook](https://www.facebook.com/groups/gardeningtipsforbeginners/)

Addicted to Gardening

[(2) Addicted To Gardening | Facebook](https://www.facebook.com/groups/addictedtogardening/)

Free Stuff Only

[(2) Facebook Marketplace | Facebook](https://www.facebook.com/marketplace/category/free/)

Free Stuff All the Time

[(2) FREE STUFF ALL THE TIME | Facebook](https://www.facebook.com/groups/1625832544402966)

East Texas Free Stuff & Helping Others in Need

[(2) East Texas FREE stuff & Helping others in need | Facebook](https://www.facebook.com/groups/1684074178549085)

Everything’s Free ~USA Only~

[(2) ~Everything's Free~ (USA ONLY) | Facebook](https://www.facebook.com/groups/EverythingsFree2012/)

Free or Cheap

[(2) Free or Cheap | Facebook](https://www.facebook.com/groups/503835309723739)

Free Farm Stuff

[(2) Free farm Stuff | Facebook](https://www.facebook.com/groups/1060242253987624)

Free Stuff Indiana

[(2) Free Stuff Indiana | Facebook](https://www.facebook.com/groups/1281493228890641)

Free Snake Relocation Directory

[(2) Free Snake Relocation Directory | Facebook](https://www.facebook.com/groups/FreeSnakeRemovalDirectory)

Free Stuff Arizona Only

[(2) Free Stuff Arizona Only! | Facebook](https://www.facebook.com/groups/320232029785407)

Free Stuff

[(2) FREE STUFF | Facebook](https://www.facebook.com/groups/299031286846832)

Date Ideas & Things to do STL

[(2) Date Ideas & Things To Do In STL | Facebook](https://www.facebook.com/groups/datersanddoersstl)

Women of Impact

[(3) Women of Impact | Facebook](https://www.facebook.com/groups/natgeowomenofimpact)

Starbucks Lovers

[(3) Starbucks Lovers ☕💚 | Facebook](https://www.facebook.com/groups/159280646036268)

Aldi Aisle of Shame

[(3) Aldi Aisle of Shame | Facebook](https://www.facebook.com/aisleofshame)

What kind of snake is this? Florida

[(3) What kind of snake is this? Florida. | Facebook](https://www.facebook.com/groups/FloridaSnakeID)

Snake Identification and Education

[(3) Snake Identification and Education | Facebook](https://www.facebook.com/groups/869432383161612)

Virginia Wild Snake ID

[(3) Virginia Wild Snake ID | Facebook](https://www.facebook.com/groups/2202245479875635)

Ethnoherpetology: Snake History & Culture

[(3) Ethnoherpetology: Snakes in History & Culture | Facebook](https://www.facebook.com/groups/309676306108765)

Home for Peculiar Artists

[(3) Home for Peculiar Artists | Facebook](https://www.facebook.com/groups/425351158406372)

The Museum of Imagination Unusual Art and Things

[(3) The Museum of Imagination ✨ Unusual Art and Things | Facebook](https://www.facebook.com/groups/186215269489623)

Online Surveys

[(3) ONLINE SURVEYS | Facebook](https://www.facebook.com/groups/173065791050365)

Dissertation Survey Exchanges

[(3) Dissertation Survey Exchange | Facebook](https://www.facebook.com/groups/ShareYourSurvey)

Survey Sharing 2021 – *Date has been changed since our survey; same site.*

[(3) Survey sharing 2022-2023 (English, Active Only) | Facebook](https://www.facebook.com/groups/surveysharing)

Survey Exchange

[(3) Survey Exchange | Facebook](https://www.facebook.com/groups/students.survey.exchange)

Dissertation Survey Exchange

[(3) Dissertation Survey Exchange | Facebook](https://www.facebook.com/groups/ShareYourSurvey)

Student Survey Exchange

[(3) Student Survey Exchange | Facebook](https://www.facebook.com/groups/225472898392397)

The Research Survey Exchange Group

[(3) The Research Survey Exchange Group | Facebook](https://www.facebook.com/groups/1376853029260212)

Student Questionnaires Survey

[(3) Students Questionnaires Survey | Facebook](https://www.facebook.com/groups/180479505786723)

­­­­­­­­­­­­­­­

­­­­­­­­­­­­­---------------------------------------------------------------------------------------------------------------------

III. Sites where Facebook Market place was used to produce participants.

The following text was advertised in the following cities Facebook Market place.

“Free IPad”

Please take this survey to be entered to win a free iPad

Potential participants were told that they would be put into a drawing for a “Free Ipad” in the following cities Facebook Marketplace:

-New York, New York

-Las Vegas, NV

-Dallas, TX

-Miami, FL

-Atlanta, GA

-Detroit, MI

-Seattle, WA

-Phoenix, AZ

---------------------------------------------------------------------------------------------------------------------

IV. Instagram hashtags used to share the Rattlesnake Survey

#OnlyGoodSnakeIsADeadSnake

#killsnakes

#deadsnake

#killrattlesnakes

#killvenomoussnakes

#rattlesnakeroundup

#snakerodeo

#snakesarebad

#badsnake

#ihatesnakes

#snakesarescary

#scarysnakes

---------------------------------------------------------------------------------------------------------------------

V. Twitter Utilization to share the Rattlesnake Survey.

Emily N. Taylor made a public Tweet on Twitter advertising participation in the Rattlesnake Survey.

VI. Reddit utilization for sharing the Rattlesnake Survey

Survey exchange subreddits were used, and shared among participants, specifically the sub reddit “Help a Graduate Student”.
