# Supplementary material for: Effects of relational and instrumental messaging on human perception of rattlesnakes
Source: PLoS One. 2024 Apr 17;19(4):e0298737. doi: 10.1371/journal.pone.0298737 (PMC11023442; doi:10.1371/journal.pone.0298737)
Supplement: S3 Text — (DOCX) [file pone.0298737.s004.docx]

**S3 Text.** **Relational Video Script.**

Rattlesnakes display social behaviors that are similar to people. At certain times of the year, rattlesnakes congregate in community dens. Historically, it was believed that the purpose of den sharing was to obtain refuge from the cold. However, in areas with relatively mild winters, rattlesnakes still gather in dens on occasion. Scientists have discovered that rattlesnakes are social creatures and have friends or family who they prefer to be close to.

At shared nesting sites, expecting mothers gather next to each other to give birth. Did you know that instead of laying eggs, females give birth to live young called pups? She looks after her litter until the pups undergo their first shed and are able to hunt on their own. Young rattlesnakes tend to stay near older, more experienced adults. Some pups will follow their parents to overwintering dens and prime basking locations.

Other mature rattlesnakes will occasionally stay near pups to protect them from dangerous predators. Foxes, kingsnakes, owls, and bobcats are examples of animals that prey on young snakes***.*** Similarly to people, rattlesnakes form relationships with friends and family and take care of their babies.
