# Supplementary material for: Effects of relational and instrumental messaging on human perception of rattlesnakes
Source: PLoS One. 2024 Apr 17;19(4):e0298737. doi: 10.1371/journal.pone.0298737 (PMC11023442; doi:10.1371/journal.pone.0298737)
Supplement: S4 Text — (DOCX) [file pone.0298737.s005.docx]

**S4 Text.** **Instrumental Video Script.**

Rattlesnakes have a positive impact on the ecosystems that they are part of, and this impact actually helps people. Rattlesnakes can live twenty-five years or more in the wild and contribute valuable services to the ecosystems in which they inhabit. They control rodent populations as their diet is largely dependent on small mammals. If left unmanaged, rodent populations can explode, causing an increase in disease spread and damage to agricultural crops and native plant communities. Rattlesnakes reduce the spread of tick-borne pathogens like Lyme disease because when consuming rats, the snakes ingest ticks, too. Did you know an  adult rattlesnake can consume between 2,500 to 4,500 ticks per year?

Rattlesnakes also aid in seed dispersal. Oftentimes, rodents store seeds in cheek pouches which can end up in a rattlesnake's digestive tract. Recent studies have shown that seeds become viable once passed through a rattlesnake's digestive system. Some seeds must pass through an animal's digestive system in order to germinate. Rattlesnakes help plants expand their range by dispersing seeds.

Although rattlesnakes are considered predators, they are also prey for a variety of animals. For example, coyotes, foxes, owls, kingsnakes, and bobcats are animals known to prey on rattlesnakes. As both predator and prey, rattlesnakes represent an important component of food webs. Rattlesnakes promote balance in their ecosystem and offer services that are of great importance to the environments in which they live.
