# Supplementary material for: Effects of relational and instrumental messaging on human perception of rattlesnakes
Source: PLoS One. 2024 Apr 17;19(4):e0298737. doi: 10.1371/journal.pone.0298737 (PMC11023442; doi:10.1371/journal.pone.0298737)
Supplement: S1 Table — (DOCX) [file pone.0298737.s006.docx]

**S1 Table. Treatment coefficient and standard errors of the difference in ARP score (post-pre) reflecting the effect of treatment on perception of rattlesnakes, based on full model averaging.**

| Independent variable | estimate | SEM | p |
| --- | --- | --- | --- |
| Intercept (Instrumental)* | 4.247 | 0.199 | < 0.001 |
| Relational* | -0.765 | 0.278 | 0.006 |
