# Supplementary material for: Effects of relational and instrumental messaging on human perception of rattlesnakes
Source: PLoS One. 2024 Apr 17;19(4):e0298737. doi: 10.1371/journal.pone.0298737 (PMC11023442; doi:10.1371/journal.pone.0298737)
Supplement: S2 Table — (DOCX) [file pone.0298737.s007.docx]

**S2 Table. The most likely model predicting the difference in pre- and post- ARP score for the relational message.**

| Model | d.f. | ll | AICc | 𝚫AICc | w |
| --- | --- | --- | --- | --- | --- |
| generation X religion X gender | 14 | -741.74 | 1513.00 | 0 | 0.80 |
| generation X gender | 8 | -749.69 | 1515.90 | 2.89 | 0.19 |
| generation X religion | 12 | -748.59 | 1522.30 | 9.30 | 0.01 |
| null | 2 | -772.80 | 1549.64 | 36.63 | 0 |
