# Supplementary material for: Effects of relational and instrumental messaging on human perception of rattlesnakes
Source: PLoS One. 2024 Apr 17;19(4):e0298737. doi: 10.1371/journal.pone.0298737 (PMC11023442; doi:10.1371/journal.pone.0298737)
Supplement: S3 Table — (DOCX) [file pone.0298737.s008.docx]

**S3 Table. Full model averaging of the effects of generation, religion, and sex on difference in Aggregate Rattlesnake Perception Score (ARP) after viewing the relational message.** Asterisks indicate statistically significant coefficients that predict the model (see p value). Intercept indicates being a baby boomer, agnostic, and female and all generation, religion, and gender coefficient estimates are compared to this intercept.

| Independent variable | estimate | SEM | p |
| --- | --- | --- | --- |
| Intercept* (baby-boomer/agnostic/female) | 3.534 | 0.803 | < 0.001 |
| Millennial | 0.406 | 0.554 | 0.408 |
| Silent generation | -1.887 | 3.185 | 0.555 |
| Generation-X | 0.549 | 0.650 | 0.401 |
| Generation-Z* | 2.490 | 0.546 | < 0.001 |
| Atheist | -1.519 | 1.031 | 0.142 |
| Buddhist | -4.672 | 3.695 | .207 |
| Christian | -0.647 | 0.672 | 0.337 |
| Muslim* | 3.943 | 2.812 | 0.163 |
| Jewish | -1.771 | 1.542 | 0.252 |
| Spiritual | -0.845 | 0.872 | 0.334 |
| Male* | -1.543 | 0.449 | < 0.001 |
| Non-binary | -1.478 | 1.630 | 0.367 |
