# Supplementary material for: Effects of relational and instrumental messaging on human perception of rattlesnakes
Source: PLoS One. 2024 Apr 17;19(4):e0298737. doi: 10.1371/journal.pone.0298737 (PMC11023442; doi:10.1371/journal.pone.0298737)
Supplement: S4 Table — (DOCX) [file pone.0298737.s009.docx]

**S4 Table. Full model averaging of the effects of generation, religion, and sex on difference in Aggregate Rattlesnake Perception Score (ARP) after viewing the instrumental message.** Asterisks indicate statistically significant generation coefficients that predict the model (see p value). Intercept indicates being agnostic and all religion coefficient estimates are compared to this intercept.

| Independent variable | estimate | SEM | p |
| --- | --- | --- | --- |
| Intercept (agnostic)* | 3.600 | 0.691 | < 0.001 |
| Atheist | -0.669 | 0.942 | 0.478 |
| Buddhist | 1.900 | 2.54 | 0.455 |
| Christian | 0.992 | 0.733 | 0.177 |
| Hindu* | 6.900 | 2.537 | 0.007 |
| Muslim* | 5.733 | 2.110 | 0.007 |
| Jewish | 1.400 | 1.859 | 0.452 |
| Spiritual | 0.088 | 1.105 | 0.937 |
