# Supplementary material for: Effects of relational and instrumental messaging on human perception of rattlesnakes
Source: PLoS One. 2024 Apr 17;19(4):e0298737. doi: 10.1371/journal.pone.0298737 (PMC11023442; doi:10.1371/journal.pone.0298737)
Supplement: S5 Table — (DOCX) [file pone.0298737.s010.docx]

**S5 Table. The most likely model predicting the effect of a snake bite experience and difference in Aggregate Rattlesnake (ARP) score.**

| Model | d.f. | ll | AICc | 𝚫AICc | w |
| --- | --- | --- | --- | --- | --- |
| Snake bite | 3 | -4196.92 | 8399.86 | 0.00 | 0.80 |
| Snake bite X treatment | 5 | -4196.28 | 8402.62 | 2.75 | 0.20 |
| null | 2 | -4212.56 | 8429.12 | 29.26 | 0.00 |
