# Supplementary material for: Effects of relational and instrumental messaging on human perception of rattlesnakes
Source: PLoS One. 2024 Apr 17;19(4):e0298737. doi: 10.1371/journal.pone.0298737 (PMC11023442; doi:10.1371/journal.pone.0298737)
Supplement: S6 Table — (DOCX) [file pone.0298737.s011.docx]

**S6 Table. Full model averaging results for the influence of snake bite experience and treatment on Aggregate Rattlesnake Perception (ARP) score.** Asterisks indicate statistically significant coefficients that predict the model (see p value). Intercept indicates not having a venomous snake bite experience and coefficient estimates are compared to this intercept.

| Independent variable | estimate | SEM | p |
| --- | --- | --- | --- |
| Intercept (No bite)* | 6.052 | 0.593 | < 0.001 |
| Yes bite* | -1.430 | 0.444 | 0.001 |
| Treatment* | -0.439 | 0.831 | 0.598 |
| Treatment x snake bite | -0.265 | 0.622 | 0.670 |
