# Supplementary material for: Effects of relational and instrumental messaging on human perception of rattlesnakes
Source: PLoS One. 2024 Apr 17;19(4):e0298737. doi: 10.1371/journal.pone.0298737 (PMC11023442; doi:10.1371/journal.pone.0298737)
Supplement: Attachment — Submitted filename: Response to Reviewers.docx [file pone.0298737.s012.docx]

**Dear Anita Mitico Tanaka-Azevedo:	Jan 15, 2024**

**Thank you for handling the review of our manuscript “Effects of relational and instrumental messaging on human perception of rattlesnakes.” We have addressed the comments of the reviewers (see responses in bold below) and edited the manuscript to conform to PLoS ONE style. Thank you,**

**The authors**

Journal Requirements:

When submitting your revision, we need you to address these additional requirements.

1. Please ensure that your manuscript meets PLOS ONE's style requirements, including those for file naming. The PLOS ONE style templates can be found at 
<https://journals.plos.org/plosone/s/file?id=wjVg/PLOSOne_formatting_sample_main_body.pdf> and 
<https://journals.plos.org/plosone/s/file?id=ba62/PLOSOne_formatting_sample_title_authors_affiliations.pdf>

**We have edited the manuscript to conform to PLoS ONE style.**

2. Did you know that depositing data in a repository is associated with up to a 25% citation advantage (<https://doi.org/10.1371/journal.pone.0230416>)? If you’ve not already done so, consider depositing your raw data in a repository to ensure your work is read, appreciated and cited by the largest possible audience. You’ll also earn an Accessible Data icon on your published paper if you deposit your data in any participating repository (<https://plos.org/open-science/open-data/#accessible-data>).

**We have deposited our data on Dryad. https://datadryad.org/stash/share/X-vR3xg0zGE7Vgo3a-9OWyZhymmYE6DMOWIfltLcK_0**

3. Please provide additional details regarding participant consent. In the ethics statement in the Methods and online submission information, please ensure that you have specified what type you obtained (for instance, written or verbal, and if verbal, how it was documented and witnessed). If your study included minors, state whether you obtained consent from parents or guardians. If the need for consent was waived by the ethics committee, please include this information.

**We edited the Ethics Statement to clearly show that no data from minors was included and to describe how we obtained informed consent.**

4. We note that your Data Availability Statement is currently as follows: "All relevant data and supporting information will be included as online supplemental material."

Please confirm at this time whether or not your submission contains all raw data required to replicate the results of your study. Authors must share the “minimal data set” for their submission. PLOS defines the minimal data set to consist of the data required to replicate all study findings reported in the article, as well as related metadata and methods (<https://journals.plos.org/plosone/s/data-availability#loc-minimal-data-set-definition>).

For example, authors should submit the following data:

- The values behind the means, standard deviations and other measures reported;
- The values used to build graphs;
- The points extracted from images for analysis.

Authors do not need to submit their entire data set if only a portion of the data was used in the reported study.

If your submission does not contain these data, please either upload them as Supporting Information files or deposit them to a stable, public repository and provide us with the relevant URLs, DOIs, or accession numbers. For a list of recommended repositories, please see <https://journals.plos.org/plosone/s/recommended-repositories>.

If there are ethical or legal restrictions on sharing a de-identified data set, please explain them in detail (e.g., data contain potentially sensitive information, data are owned by a third-party organization, etc.) and who has imposed them (e.g., an ethics committee). Please also provide contact information for a data access committee, ethics committee, or other institutional body to which data requests may be sent. If data are owned by a third party, please indicate how others may request data access.

**See above- data are now on Dryad.**

5. When completing the data availability statement of the submission form, you indicated that you will make your data available on acceptance. We strongly recommend all authors decide on a data sharing plan before acceptance, as the process can be lengthy and hold up publication timelines. Please note that, though access restrictions are acceptable now, your entire data will need to be made freely accessible if your manuscript is accepted for publication. This policy applies to all data except where public deposition would breach compliance with the protocol approved by your research ethics board. If you are unable to adhere to our open data policy, please kindly revise your statement to explain your reasoning and we will seek the editor's input on an exemption. Please be assured that, once you have provided your new statement, the assessment of your exemption will not hold up the peer review process.

**See above- data are now on Dryad.**

6. Please include captions for your Supporting Information files at the end of your manuscript, and update any in-text citations to match accordingly. Please see our Supporting Information guidelines for more information: <http://journals.plos.org/plosone/s/supporting-information>.

**We have added captions for supplemental information at the end of our manuscript.**

[Note: HTML markup is below. Please do not edit.]

Reviewers' comments:

Reviewer's Responses to Questions

Comments to the Author

1. Is the manuscript technically sound, and do the data support the conclusions?

The manuscript must describe a technically sound piece of scientific research with data that supports the conclusions. Experiments must have been conducted rigorously, with appropriate controls, replication, and sample sizes. The conclusions must be drawn appropriately based on the data presented.

Reviewer #1: Yes

Reviewer #2: Partly

2. Has the statistical analysis been performed appropriately and rigorously?

Reviewer #1: Yes

Reviewer #2: Yes

3. Have the authors made all data underlying the findings in their manuscript fully available?

The [PLOS Data policy](http://www.plosone.org/static/policies.action#sharing) requires authors to make all data underlying the findings described in their manuscript fully available without restriction, with rare exception (please refer to the Data Availability Statement in the manuscript PDF file). The data should be provided as part of the manuscript or its supporting information, or deposited to a public repository. For example, in addition to summary statistics, the data points behind means, medians and variance measures should be available. If there are restrictions on publicly sharing data—e.g. participant privacy or use of data from a third party—those must be specified.

Reviewer #1: Yes

Reviewer #2: Yes

4. Is the manuscript presented in an intelligible fashion and written in standard English?

PLOS ONE does not copyedit accepted manuscripts, so the language in submitted articles must be clear, correct, and unambiguous. Any typographical or grammatical errors should be corrected at revision, so please note any specific errors here.

Reviewer #1: Yes

Reviewer #2: Yes

5. Review Comments to the Author

Please use the space provided to explain your answers to the questions above. You may also include additional comments for the author, including concerns about dual publication, research ethics, or publication ethics. (Please upload your review as an attachment if it exceeds 20,000 characters)

Reviewer #1: The study is very interesting and can help in the conservation of non-empathetic animals, through simple message framing methods targeted at the population. The comparison of results according to social,gender and age variables are also very newsworthy. The study was carried out in an efficient way, with interesting results and conclusions.

Reviewer #2: The manuscript by Allison et al. aims to test the effects of relational and instrumental message strategies on US residents’ perception of rattlesnakes, through deploying online survey to social media users (n=1,182) to describe perceptions of rattlesnakes and assess the change after viewing a randomly selected relational or instrumental video message. Specifically, authors investigated: 1) How do socio–demographic factors and prior experience with a venomous snake bite affect participants’ baseline perceptions of rattlesnakes? 2) How do the instrumental and relational message strategies change participants’ perception of rattlesnakes? 3) How do socio–demographic factors and prior experience with a venomous snake bite influence the impacts of relational or instrumental message strategies on perception of rattlesnakes?
The study seems to me to be very effective in mapping different people's perceptions of rattlesnakes, and even in measuring the effectiveness of changing these perceptions following new information about the species among residential categories, geographic region, generation, religion, and gender. However, there are some important issues to consider. The main problem observed is the subjective nature of the importance of the relational or instrumental method in the efficiency of changing the perception of the target audience. I find it difficult to disentangle the effect that the videos of each approach have on people's individual preferences. How do we know if the effectiveness of the videos is associated with a particular approach or just with the script, filming, selection of images, etc.? Could there be significant differences between different videos within the same approach, relational or instrumental?

**We thank the reviewer for drawing attention to this issue. While it is true that different images and language in each video could have acted as confounding variables to influence the results, for the most part the content was matched to the messaging. We have added the following passage to the Discussion to acknowledge this issue:**

**Although the videos were similar in length and had the same narrator, each video had different content (video footage and script), making it possible that one or more aspects of the content could have contributed to respondents’ changes in perception in unintended ways. For example, a respondent might have found the footage showing rattlesnakes eating rodents in the instrumental video to be repulsive. We acknowledge that such content differences could confound our interpretation of the results as purely relational versus instrumental. However, for the most part, the content differences between the videos were explicitly associated with the intended messaging. For example, we showed rattlesnake mothers and babies, and used words like “friends” and “family,” in the script of the relational but not the instrumental messaging video as part of an intentional effort to show values with which people might relate.**

I also believe that the authors could review their decision to consider as "No" those participants who answered "Not sure" to the question "you, a friend, or your pet was bitten by a venomous snake.". It would make more sense to consider this group separately in the analyses, or even to exclude these individuals from the sample, so as not to compromise the reliability of the results by adding uncertainties to the model.

**We have now removed individuals from the analysis who answered “Not Sure” for this question. Removal of these individuals did not change the results, but (unsurprisingly) did change the delta AIC values and mean values of pre-ARP scores for individuals who answered “yes” or “no” to this question. This information is now updated in our manuscript and Figure 3.**

Graham, Z. is listed as corresponding author in PLOSOne system, but Loughman, Z.J. is marked with * on the first page. Please, check who should be the correct correspondent author.

**Loughman Z.J. is the corresponding author, but this manuscript is being submitted through Graham, Z. in correspondence with Loughman, Z.J.**

6. PLOS authors have the option to publish the peer review history of their article ([what does this mean?](https://journals.plos.org/plosone/s/editorial-and-peer-review-process#loc-peer-review-history)). If published, this will include your full peer review and any attached files.

If you choose “no”, your identity will remain anonymous but your review may still be made public.

Do you want your identity to be public for this peer review? For information about this choice, including consent withdrawal, please see our [Privacy Policy](https://www.plos.org/privacy-policy).

Reviewer #1: No

Reviewer #2: No
